# Influence of Extremely High Pressure and Oxygen on Hydrocarbon-Enriched Microbial Communities in Sediments from the Challenger Deep, Mariana Trench

**DOI:** 10.3390/microorganisms11030630

**Published:** 2023-03-01

**Authors:** Ying Liu, Songze Chen, Zhe Xie, Li Zhang, Jiahua Wang, Jiasong Fang

**Affiliations:** 1 Shanghai Engineering Research Center of Hadal Science and Technology, Shanghai Ocean University, Shanghai 200120, China; 2 Shenzhen Key Laboratory of Marine Archaea Geo-Omics, Department of Ocean Science and Engineering, Southern University of Science and Technology, Shenzhen 518000, China; 3 Laboratory for Marine Biology and Biotechnology, Qingdao National Laboratory for Marine Science and Technology, Qingdao 266000, China; 4 Department of Natural Sciences, Hawaii Pacific University, Honolulu, HI 96813, USA

**Keywords:** *n*-alkane-enriched microorganisms, Mariana Trench sediments, hydrostatic pressure, anaerobic and aerobic incubation, short-chain and long-chain *n*-alkanes, long-period culture

## Abstract

Recent studies reported that highly abundant alkane content exists in the ~11,000 m sediment of the Mariana Trench, and a few key alkane-degrading bacteria were identified in the Mariana Trench. At present, most of the studies on microbes for degrading hydrocarbons were performed mainly at atmospheric pressure (0.1 MPa) and room temperature; little is known about which microbes could be enriched with the addition of *n*-alkanes under in-situ environmental pressure and temperature conditions in the hadal zone. In this study, we conducted microbial enrichments of sediment from the Mariana Trench with short-chain (SCAs, C_7_–C_17_) or long-chain (LCAs, C_18_–C_36_) *n*-alkanes and incubated them at 0.1 MPa/100 MPa and 4 °C under aerobic or anaerobic conditions for 150 days. Microbial diversity analysis showed that a higher microbial diversity was observed at 100 MPa than at 0.1 MPa, irrespective of whether SCAs or LCAs were added. Non-metric multidimensional scaling (nMDS) and hierarchical cluster analysis revealed that different microbial clusters were formed according to hydrostatic pressure and oxygen. Significantly different microbial communities were formed according to pressure or oxygen (*p* < 0.05). For example, Gammaproteobacteria (*Thalassolituus*) were the most abundant anaerobic *n*-alkanes-enriched microbes at 0.1 MPa, whereas the microbial communities shifted to dominance by Gammaproteobacteria (*Idiomarina*, *Halomonas*, and *Methylophaga*) and Bacteroidetes (*Arenibacter*) at 100 MPa. Compared to the anaerobic treatments, Actinobacteria (*Microbacterium*) and Alphaproteobacteria (*Sulfitobacter* and *Phenylobacterium*) were the most abundant groups with the addition of hydrocarbon under aerobic conditions at 100 MPa. Our results revealed that unique *n*-alkane-enriched microorganisms were present in the deepest sediment of the Mariana Trench, which may imply that extremely high hydrostatic pressure (100 MPa) and oxygen dramatically affected the processes of microbial-mediated alkane utilization.

## 1. Introduction

Hydrocarbons are ubiquitous in the oceans and are mainly derived from natural seepage and oil spills [1,2,3]. Alkanes, which are divided into *n*-alkanes, branched-chain alkanes, and cycloalkanes, are major constituents of petroleum [4]. A range of alkanes is produced by plants, green algae, bacteria, and animals [5,6]. In particular, marine cyanobacteria were reported to produce and accumulate hydrocarbons (mainly C_15_ and C_17_ alkanes), with a global ocean hydrocarbon production of ∼308–771 million tons annually [2,6]. 

Microbes from all three domains of life can utilize hydrocarbons as the sole source of carbon and energy [7,8]. A total of at least 175 prokaryotic genera are known to degrade hydrocarbons under aerobic or anaerobic conditions [9]. For example, hydrocarbon-degrading bacteria contained *Alcanivorax*, *Cycloclasticus*, *Marinobacter*, *Oleispira*, etc.; hydrocarbon-degrading fungi mainly consisted of *Aspergillus*, *Mucor*, *Fusarium*, and *Penicilium* [5,8,10,11,12,13,14,15]. The degradation mechanism of alkanes is that most alkane-degrading bacteria secrete diverse surfactants that facilitate emulsification and uptake of the alkanes [5,16].

Many environmental factors, such as hydrostatic pressure, oxygen, and hydrocarbon components, affect hydrocarbon biodegradation [17]. A series of accumulating findings revealed that high hydrostatic pressure (HHP) has an important influence on hydrocarbon biodegradation in deep-sea environments [7,18,19,20,21,22,23,24,25,26]. First, high pressure inhibited the growth of hydrocarbon-degrading bacteria [20,22,27,28,29]. For example, the bacterial growth rates and yields for *n*-hexadecane utilization at in situ temperatures (4 °C) and high pressure (50 MPa) in the deep ocean were much lower as compared to the incubations conducted at 0.1 MPa and 4 °C [20]. The growth and activity of hydrocarbon-degrading *Alcanivorax borkumensis* were impaired at <10 MPa [27]. The growth of strain *Sphingobium yanoikuyae* in the degradation of aromatic hydrocarbons was severely inhibited at 8 MPa [22]. The growth of *Rhodococcus* isolated from deep-sea sediment is impaired at 15 MPa [29]. Second, a 4% decrease in *n*-alkane biodegradation was observed for every 1 MPa increase [28]. Finally, high pressure is also known to affect the succession of microbial communities in hydrocarbon degradation [19,25,30]. For instance, members of the genus *Sulfitobacter* were highly abundant in hydrocarbon degradation at 0.1 MPa, whereas *Photobacterium* dominated the hydrocarbon-degrading microbial communities at 30 MPa [25].

It has been reported that different components of hydrocarbon were primary drivers for selecting well-adapted specific microbial communities [31,32]. Microbial community preferentially utilized oil-derived short-chain and higher-molecular weight alkanes [33,34]. Several studies indicated that *Colwellia* had the capability to oxidize ethane, propane, and butane [35], *Oceanospirillum* could degrade cyclohexane [36], and *Cycloclasticus* could utilize BTEX (benzene, toluene, ethylbenzene, and xylenes) as well as polycyclic aromatic hydrocarbons (PAHs) [9]. 

Microorganisms could degrade all kinds of hydrocarbons under both aerobic and anaerobic conditions [37,38]. The processes of aerobic hydrocarbon degradation generally occurred throughout the marine water column and oxic surface sediments even in deep waters [39], whereas anaerobic hydrocarbon degradation primarily existed in anoxic bottom sediments and within hydrocarbon seeps [40,41]. Aerobic hydrocarbon degradation requires oxygen as a reactant for alkane activation. The aerobic oxidation of *n*-alkanes can be catalyzed by different enzymes, including methane monooxygenases [42], cytochrome P450, integral membrane non-heme iron monooxygenases (named AlkB) [5,43], flavin-binding monooxygenase (named AlmA) [44], and a long-chain alkane hydroxylase (named LadA) [45]. The aerobic microorganisms for hydrocarbon degradation mainly contained *Bacillus* spp., *Acinetobacter* spp., and *Pseudomonas* spp. [37]. On the contrary, anaerobic degradation of hydrocarbons has been shown to occur with Fe^3+^, SO_4_^2−^ and NO_3_^−^ as electron acceptors [4,46]. The anaerobic degradation rate of hydrocarbons is usually shown to be several orders of magnitude lower, and the related genes and enzymes involved in these pathways are less known [47]. At present, the mainly anaerobic alkane-degraders are affiliated with members of the Proteobacteria (Gamma-, Delta-, and Epsilon-), Firmicutes, and Euryarchaeota [41,48,49]. However, most studies focused on soil, freshwater, shorelines, and surface water contamination at atmospheric pressure; there are few studies involved in anaerobic alkane degradation under high pressure [40,50,51].

The ocean’s hadal zone (depth > 6000 m) is characterized by extreme physical-chemical conditions, including high hydrostatic pressure (HHP, ~110 MPa), low temperature (LT, 1.0–2.5 °C), low dissolved oxygen (156 μM), and lack of sunlight [52,53,54]. All these factors can have a crucial influence on microbial processes and therefore further affect the rate and extent of hydrocarbon biodegradation in the hadal zone [18,19]. The latest findings revealed that abundant *n*-alkanes were detected in the surface sediments of the Mariana Trench, with total concentrations of *n*-alkanes ranging from 0.341 to 3.465 μg/g dry weight (dw) [14,55]. These *n*-alkanes were mainly derived from bacteria or marine algae in the euphotic zone and subsequently exported to the deeper waters and hadal zones of the Mariana Trench [14]. The other relevant studies reported that the abundance and expression of alkane degradation genes, such as those encoding medium-chain alkane 1-monooxygenase (AlkB) and long-chain alkane monooxygenase (AlmA), tended to increase in the bottom waters of the Mariana Trench [14]. However, the key microorganisms truly responsible for *n*-alkane degradation still unknown in the deepest habitat on earth (e.g., the Mariana Trench, 11,000 m).

Up to now, most investigations on hydrocarbon degradation were conducted at atmospheric pressure (0.1 MPa) [56,57,58,59,60], and only a few studies focused on the effect of high hydrostatic pressure, oxygen, and *n*-alkane components on alkane enrichments (the highest simulated pressure was up to 60 MPa) [14], and the corresponding results may not apply to the hadal zone of the Mariana Trench. To understand how each of the three factors (pressure, oxygen, and *n*-alkanes) affected microbial diversity and community composition in processes of aerobic and anaerobic hydrocarbon input under in-situ conditions in MT, simulation investigations in the lab were conducted. Our goals are to (1) compare the differences of microbial communities with hydrocarbon addition at 0.1 or 100 MPa, and evaluate the effect of pressure on microbial communities for hydrocarbon utilization; (2) compare the differences of the microbial groups responsible for aerobic and anaerobic alkane treatments at in situ conditions, and assess the effect of oxygen on microbial communities for hydrocarbon enrichments; (3) compare the differences of microbial communities involved in different alkane (C_7–17_ or C_18_–C_36_) addition, and assess the effect of alkane components on microbial communities for hydrocarbon utilization.

## 2. Materials and Methods 

### 2.1. Sediments Sampling 

Sediment samples were collected from the Challenger Deep of the Mariana Trench (11.329° N, 142.198° E, water depth of 10,898 m) during the cruise from 2019.12 to 2020.1. A box corer (with a base area of 400 cm^2^ and a height of 25 cm) attached to the Hadal Lander II was used to collect sediment samples from the trench [61]. Briefly, two hours after the lander touched down on the ocean floor, the box corer was slowly lowered into the seafloor until it was about 25 cm below the sediment surface. The box corer was then sealed with a lid, and the lander’s cover was put back on. The sediment samples were quickly resampled using sterile plastic corers after being recovered on board, and they were kept at 4 °C until further analysis.

### 2.2. Enrichments of n-Alkanes Experiments under High-Pressure

Sediment samples (10,898 m) were enriched using sterile LMO medium (i.e., 26 g NaCl, 5 g MgCl_2_·6H_2_O, 1.4 g CaCl_2_·2H_2_O, 4 g Na_2_SO_4_, 0.3 g NH_4_Cl, 0.1 g KH_2_PO_4_, 0.96 g KCl, 20 mM NaHCO_3_, and 4 mL of vitamin mixtures dissolved in 1 L ddH_2_O, pH 7~8) with short-chain *n*-alkanes (SCAs, C_7_–C_17_) or long-chain *n*-alkanes (LCAs, C_18_–C_36_) mixtures as a sole carbon source, and then incubated at 0.1 MPa/100 MPa and 4 °C for about 150 days in the dark. 

A total of five incubation treatments were conducted in our study. Firstly, anaerobic enrichment cultures of SCAs or LCAs consisted of 100 g of sediment, 20 mL of sterile LMO medium supplemented with 120 μL of the C_7_–C_17_ mixtures (1:1, *v*/*v*, 1 mL/L final concentration), or 0.12 g of the C_18_–C_36_ mixtures (1:1, *w*/*w*, 1 g/L final concentration) at 0.1 MPa or 100 MPa and 4 °C for 7, 18, 30, and 11, 21, 30, 150 days, respectively. All these components were injected into a sterile anaerobic culture bag with the addition of resazurin solution (1 g/L) as a redox indicator. Finally, all the anaerobic culture bags needed to be oxygen-free before incubation. Triplicate incubations were done for all treatments. 

On the other hand, aerobic enrichments of SCAs or LCAs comprised 100 g of sediments, 20 mL of LMO medium supplemented with 120 μL of C_7_–C_17_ mixture (or 0.12 g of C_18_–C_36_ mixture), and 40 mL (25% of the total volume) of oxygenated-saturated Fluorinert^TM^ (3M^TM^ Corp., Minneapolis, MN, USA) [62] at 100 MPa and 4 °C for 150 days. All incubation components were placed in a sterile pouch (Kapak SealPak 500, 120 mL) and then sealed with a heat sealer. Triplicate incubations were done for all treatments.

Then, these high-pressure bags were incubated in several cylindrical, stain-less steel pressure vessels (Feiyu Petrochemical Instrument Equipment Inc., Nantong Guilin, China), with a maximum working pressure of 120 MPa. Hydrostatic pressure was created by pumping water using a hand-operated pump. 

### 2.3. DNA Extraction 

The genomic DNA from the cultures was extracted with the FastDNA Spin Kit for Soil (MP Biomedical, Solon, OH, USA) according to the manufacturer’s protocols. The obtained total DNA was dissolved in 100 μL DNase-free ddH_2_O. The DNA concentration was determined by Nanodrop 2000 (Thermo Scientific, Wilmington, DE, USA) and stored at −80 °C until further analysis.

### 2.4. PCR Amplification of 16S rRNA Gene

The genomic DNA of each sample was amplified using the barcoded primers 515F (5′-GTGYCAGCMGCCGCGGTAA-3′) and 806R (5′-GGACTACNVGGGTWTCTAAT-3′) [63], aiming at the V4 region of the 16S rRNA gene. The 25 μL PCR system included 10 μL Premix Taq^TM^ (Takara, Dalian, China), 0.5 μL of each primer (10 μM), about 10 ng of DNA, and 13 μL DNase-free ddH_2_O. The PCR was conducted on a thermocycler PCR system (GeneAmp 9700, Applied Biosystems) with the following program: 94 °C for 3 min; 35 cycles of 94 °C for 45 s, 50 °C for 60 s, and 72 °C for 90 s; 72 °C for 10 min. Negative and positive controls were also conducted in each run. 

### 2.5. Illumina Miseq Sequencing and Data Processing

The amplicon samples were pooled in equimolar amounts and sequenced with an Illumina MiSeq platform (Illumina, San Diego, CA, USA) at the Majorbio Bio-Pharm Technology Co., Ltd. (Shanghai, China). The raw sequences were trimmed with cutadapt (sequences with average quality scores lower than 20 were filtered, and primer sequences were cut off) [64]. The trimmed sequences were truncated, denoised, and filtered for chimeras using DADA2 commands. The amplicon sequence variants (ASVs) of 16S rRNA sequences were then classified with the classify-sklearn plugin against the SILVA 138.1 database in QIIME2 [65]. Finally, the mitochondria and unclassified ASVs were removed. These processes produced 25,181–258,284 reads of 55 samples in the ASV table, and the sequences were resampled to the minimum number of sequences (25,181) among all samples. 

### 2.6. Statistical and Ecological Analyses

The non-metric multidimensional scaling (nMDS) and hierarchical cluster analyses were performed using the PRIMER v7 package [66]. Linear discriminant analysis (LDA; http://huttenhower.sph.harvard.edu/galaxy/root?tool_id=PICRUSt_normalize, accessed on 20 Feburary 2023) was used to identify potential biomarkers at the genus level, with an LDA score threshold of 5.0. The phylogenetic tree was constructed using the MEGA v11.0 software [67], and the heatmap was visualized by TBtools software [68].

## 3. Results

### 3.1. Analysis of Microbial Diversity 

High-throughput sequencing (Illumina MiSeq) of the V4 region of the 16S rRNA gene resulted in up to 3,340,943 high-quality sequences, and a total of 1567 amplicon sequence variants (ASVs) were identified from 55 samples (Tables S1 and S2). The sequencing data were rarified to 25,181 reads per sample based on the lowest number of sequences, and the number of ASVs varied from 17 (SCAs01-30d-1) to 408 (SCAs100-150d-2) (Tables S1 and S2). The Good’s coverage ranged from 99.8% to 100% (Table S2), suggesting that the sequencing depth was reasonable and the diversities of the microbial community were well covered. 

Generally, the Shannon index tended to decrease with incubation time at 0.1 MPa, whereas the Shannon index remained nearly constant at 100 MPa (Figure S1 and Table S2). 

A higher Shannon diversity index was observed at high-pressure (100 MPa) than that at atmospheric pressure (0.1 MPa) whether it’s short-chain alkanes (SCAs, C_7_~C_17_) or long-chain alkanes (LCAs, C_18_~C_36_) enrichments. For instance, the value of the Shannon index at 0.1 MPa ranged from 0.8–3.3, whereas the corresponding values were 2.2–3.9 at 100 MPa (Figure S1 and Table S2).

On the other hand, the Shannon index of SCA treatments was always higher than that of LCA treatments, irrespective of pressure and oxygen. For example, the value of the Shannon index in anaerobic SCA treatments (1.3–3.9) was higher than that of LCA treatments (0.8–2.7 a) at 0.1 MPa and 100 MPa. Interestingly, the Shannon index for aerobic SCA enrichments (2.8) was higher than that of LCAs (2.6) at 100 MPa (Figure S1 and Table S2). In addition, comparing anaerobic with aerobic enrichments, the Shannon index of SCAs100-150d (3.6) was much higher than that of SCAs100-150d-ae (2.8). However, much lower diversity was observed at LCAs100-150d (2.3) than that of LCAs-150d- ae (2.6) (Figure S1 and Table S2).

**Figure 1:**
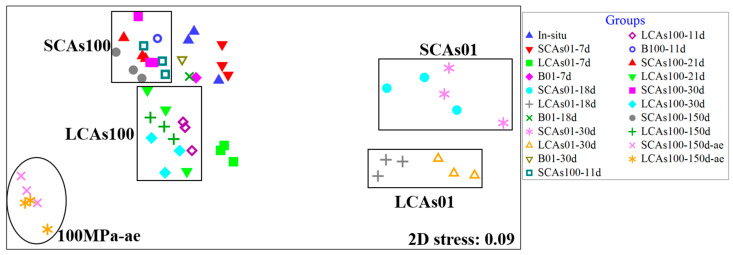
The non-metric multidimensional scaling (nMDS) analysis of microbial communities for aerobic and anaerobic hydrocarbon incubation at 0.1 and 100 MPa in the sediments of the Mariana Trench. Note: SCAs100, MT sediments added short-chain alkanes incubated anaerobically at 100 MPa; LCAs100, MT sediments added long-chain alkanes incubated anaerobically at 100 MPa; SCAs01, MT sediments added short-chain alkanes incubated anaerobically at 0.1 MPa; LCAs01, MT sediments added long-chain alkanes incubated anaerobically at 0.1 MPa; 100 MPa-ae, MT sediments added short-chain or long-chain alkanes incubated aerobically at 100 MPa.

The nMDS analysis was performed to evaluate the similarities of microbial communities according to different pressures, oxygen levels, and *n*-alkane components. A total of five clusters were formed: the SCA01, LCAs01, SCAs100, LCAs100, and 100 MPa-ae clusters (Figure 1).

Hierarchical cluster analysis (Figure S2) also revealed five distinct sample branches according to the hydrostatic pressure: two branches included the sediments incubated with SCAs or LCAs at 0.1 MPa, and another two branches contained the sediments incubated with SCAs or LCAs at 100 MPa. On the other hand, according to oxygen availability, the fifth branch included aerobic SCAs or LCAs-enriched samples at 100 MPa. Notably, the in-situ samples were clustered closely with the SCAs-100 samples.

### 3.2. Microbial Community Structure

**Figure 2:**
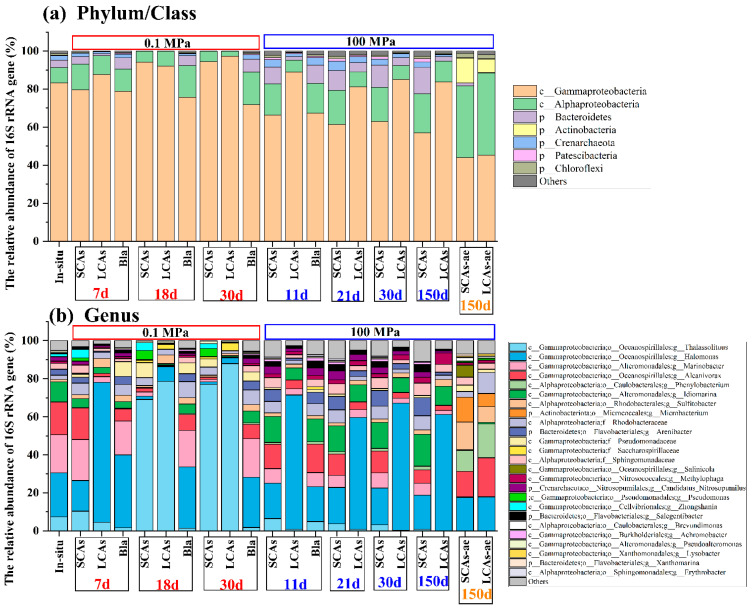
The most abundant microbial communities in the aerobic and anaerobic hydrocarbon enrichments under 0.1 and 100 MPa conditions at phylum (**a**) and genus (**b**) levels, respectively. Note: SCAs, short-chain alkanes; LCAs, long-chain alkanes; SCAs-ae: MT sediments with short-chain alkanes incubated aerobically; LCAs-ae: MT sediments with long-chain alkanes incubated aerobically; Bla, Blank.

Microbial community analysis (Figure 2) showed that different compositions of the microbial community were identified among the three different incubation treatments (0.1 MPa vs. 100 MPa; anaerobic vs. aerobic; SCAs vs. LCAs) after 150 days of incubation. 

Before incubation, the in-situ microbial communities at the phylum/class level were dominated by Gammaproteobacteria (83.2% of the total reads), Alphaproteobacteria (7.9%), Bacteroidetes (4.1%), and Crenarchaeota (2.3%) (Figure 2a). The dominant in-situ microbial genera mainly included *Halomonas* (23.1%), *Marinobacter* (20.2%), and *Alcanivorax* (17.1%) (Figure 2b). 

After incubation, the dominant microbial community in anaerobic *n*-alkane treatments at 0.1 MPa was significantly different from the corresponding treatments at 100 MPa (*p* < 0.05). 

For example, at 0.1 MPa, Gammaproteobacteria tended to increase with incubation time, with the average relative abundance changing from 79.7% (or 87.7%) at 7 d to 94.4% (or 97.2%) at 30 d in anaerobic SCAs (or LCAs) treatments. However, Alphaproteobacteria seemed to decrease with incubation time, with the average relative abundance varying from 13.3% (or 9.7%) at 7 d to 5.5% (or 2.6%) at 30 d in anaerobic SCA (or LCA) treatments. In addition, Bacteroidetes appeared to decrease dramatically in both SCAs and LCAs treatments, with 0.1% of the average relative abundance (Figure 2a).

On the contrary, at 100 MPa, Both Alphaproteobacteria and Bacteroidetes increased with the incubation period in SCAs and LCAs microcosms under anaerobic conditions. The relative abundance of Alphaproteobacteria elevated slowly from 16.3% (and 6.4%) at 11 d to 20.4% (and 10.9%) at 150 d in SCA and LCA enrichments. Similarly, the relative abundance of Bacteroidetes increased from 8.8% (and 1.7%) at 11 d to 13.9% (and 2.2%) at 150 d in SCA and LCA treatments. Nevertheless, the average relative abundance of Gammaproteobacteria decreased gradually from 66.3% (and 88.9%) at 11d to 57.1% (and 83.8%) at 150d under SCAs (and LCAs) incubation (Figure 2a).

On the other hand, the microbial communities for aerobic SCA or LCA enrichments at 100 MPa were obviously different from those of anaerobic treatments. For instance, the microbial communities in the aerobic SCAs or LCAs treatments at 100 MPa mainly consisted of Gammaproteobacteria (44.0% or 45.4%), Alphaproteobacteria (37.7% or 43.0%), and Actinobacteria (13.1% or 7.0%). The relative proportion of Gammaproteobacteria decreased sharply, whereas Alphaproteobacteria and Actinobacteria obviously increased their relative abundance in aerobic treatments compared to the in-situ samples (Figure 2a). 

**Figure 3:**
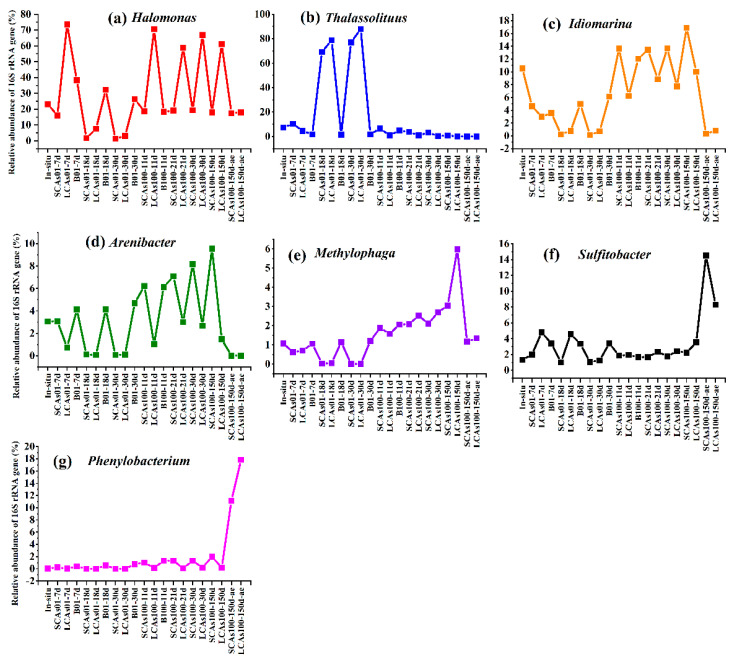
The top 7 genera in microbial communities in aerobic and anaerobic hydrocarbon enrichments at 0.1 and 100 MPa after incubated for 150 days. (**a**) The variations of relative abundance in *Halomonas* genus among different treatments and incubation time; (**b**) The variations of relative abundance in *Thalassolituus* genus among different treatments and incubation time; (**c**) The variations of relative abundance in *Idiomarina* genus among different treatments and incubation time; (**d**) The variations of relative abundance in *Arenibacter* genus among different treatments and incubation time; (**e**) The variations of relative abundance in *Methylophaga* genus among different treatments and incubation time; (**f**) The variations of relative abundance in *Sulfitobacter* genus among different treatments and incubation time; (**g**) The variations of relative abundance in *Phenylobacterium* genus among different treatments and incubation time.

At the genus level, *Thalassolituus* was the predominant microbial taxon for SCAs (or LCAs) enrichments at 0.1 MPa, with the relative abundance obviously increasing from 10.4% (or 4.5%) at 7 d to 77.1% (or 87.9%) at 30 d (Figure 2b and Figure 3). 

However, at 100 MPa, the dominant microbial genera for anaerobic SCA enrichments rapidly changed into *Idiomarina* and *Arenibacter*, with the relative abundance increasing from 13.6% and 6.2% at 11 d to 16.9% and 9.6% at 150 d (Figure 2b and Figure 3). Differently, the microbial communities for anaerobic LCAs100 enrichments were predominated by *Halomonas* (changed from 70.6% at 11 d to 61.2% at 150 d), *Idiomarina* (6.2–10.0%), and *Methylophaga* (1.6–6.0%).

In addition, under the aerobic condition at 100 MPa, key microorganisms for SCA or LCA treatments were further converted to *Sulfitobacter* (14.5 or 8.3%), *Microbacterium* (13.0 or 6.9%), *Phenylobacterium* (11.1 or 17.9%), and *Salinicola* (6.2 or 0.2%) (Figure 2b and Figure 3).

### 3.3. LDA Analysis

**Figure 4:**
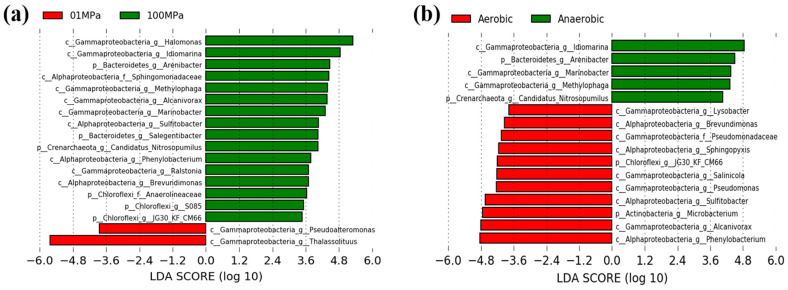
The linear discriminant analysis (LDA) identified the significantly different indicators between different pressure or oxygen treatments, with LDA scores of 5.0. (**a**) 0.1 MPa vs. 100 MPa; (**b**) aerobic vs. anaerobic.

LDA analysis (Figure 4) was used to identify the significantly different microbial indicators (with an LDA score threshold of 5.0, *p* < 0.05) that were responsible for the dissimilarities between the microbial communities for SCA or LCA enrichments under different hydrostatic pressure or oxygen conditions. 

Our results showed that a total of 2 and 16 indicative phylotypes were detected when comparing 0.1 MPa and 100 MPa, respectively (Figure 4a). For instance, two key genera, *Thalassolituus* (Gammaproteobacteria) and *Pseudoalteromonas* (Gammaproteobacteria), were responsible for the community differences at 0.1 MPa, whereas 16 dominant taxa, such as *Halomonas* (Gammaproteobacteria), *Idiomarina* (Gammaproteobacteria), *Arenibacter* (Bacteroidetes), and Sphingomonadaceae (Alphaproteobacteria) took charge of the community discrepancy at 100 MPa.

On the other hand, a total of 11 and 5 indicative microorganisms were observed when comparing aerobic and anaerobic conditions (Figure 4b). For example, *Phenylobacterium*, *Alcanivorax*, and *Microbacterium* were the main microbial indicators in aerobic treatments, whereas *Idiomarina*, *Arenibacter*, and *Marinobacter* contributed to the community differences for anaerobic hydrocarbon enrichments. 

### 3.4. Phylogenetic Analysis

**Figure 5:**
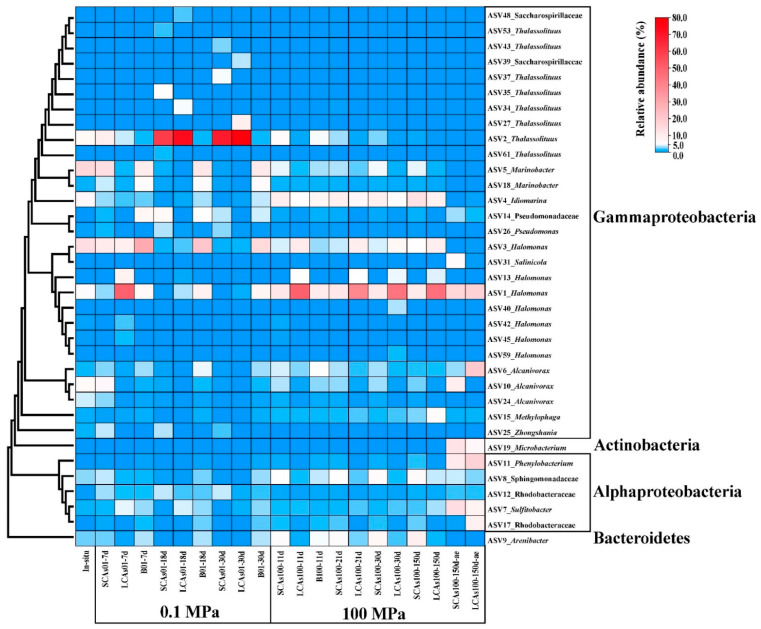
The phylogenetic tree of the 35 most abundant ASVs and 16 genera and the corresponding relative abundance are listed in the heatmap at the right.

At the ASV level, a total of 35 representative ASVs (more than 5% of the relative abundance) were chosen to construct the phylogenetic tree, and the corresponding relative abundances of the ASVs were also shown in the heatmap (Figure 5). The phylogenetic analysis revealed that these dominant microbes belonged to the three phyla Gammaproteobacteria, Alphaproteobacteria, Bacteroidetes, and Actinobacteria. 

Our results showed that a total of eight ASVs assigned to *Thalassolituus* were the most abundant taxon and responsible for anaerobic SCA and LCA enrichments at 0.1 MPa, with the relative abundance dramatically reaching 69.0% and 78.0% at 30 d. In contrast, *Idiomarina* (ASV4) dominated the anaerobic SCAs microcosm at 100 MPa, with the relative abundance increasing from 9.8% at 11 d to 13.4% at 150 d, whereas *Halomonas* (ASV1) was responsible for the anaerobic LCA enrichments at 100 MPa, with the relative abundance reaching 46.3% at 150 d. In addition, *Sulfitobacter* (ASV7), *Microbacterium* (ASV19), and *Phenylobacterium* (ASV11) were the most important species in the aerobic SCAs incubation at 100 MPa, with the relative abundance reaching 14.5%, 13.0%, and 11.1%, respectively. *Alcanivorax* (ASV6), *Phenylobacterium* (ASV11), and *Halomonas* (ASV1) predominated the aerobic LCA treatments at 100 MPa, with a relative abundance of 20.4%, 17.8%, and 17.6%, respectively (Figure 5).

## 4. Discussion 

### 4.1. Effect of Hydrostatic Pressure on Microbial Diversity and Community Composition in n-Alkane Enrichment Cultures

High pressure has an important influence on hydrocarbon biodegradation in deep-sea environments [7,19,20,22,23,24,25,26]. For instance, high pressure inhibited the growth of hydrocarbon-degrading bacteria [20,22,24,28,29], inhibited *n*-alkane biodegradation [28,29], and affected the succession of the microbial communities in the hydrocarbon enrichments [19,25,30]. 

In this study, microbial diversity for hydrocarbon enrichments at 100 MPa was obviously higher than that at 0.1 MPa (Figure S1 and Table S2), suggesting that the hydrostatic pressure had an important influence on microbial diversity with the addition of *n*-alkanes. One possible explanation is that the few microorganisms were significantly dominated at 0.1 MPa over time, resulting in a decrease in total bacterial diversity [69]. Luis and colleagues studied the effects of pressure on hydrocarbon-degrading microbial communities in subarctic sediments and also revealed that the Shannon index at 0.1 MPa was lower than that of 30 MPa (Luis et al., 2018). However, another similar investigation reported that the alpha diversities (such as Simpson and Shannon indices) of the sediment communities treated with oil at 10 MPa were lower than those of 0.1 MPa incubations (Noirungsee et al., 2020). In all, the microbial diversity associated with hydrocarbon incubation was mainly dependent on the combined effect of various environmental conditions, including pressure, temperature, oil, dispersant, etc. (Luis et al., 2018; Noirungsee et al., 2020). 

The nMDS (Figure 1) and hierarchical cluster analysis (Figure S2) commonly revealed that the microbial community was divided into two distinct clusters, i.e., 0.1 MPa-incubated and 100 MPa-treated groups, according to the hydrostatic pressure, suggesting an obvious shift of *n*-alkanes-enriched microbial communities influenced by high pressure. These results are consistent with previous similar studies conducted on subarctic sediments (Luis et al., 2018) and sediments from the Gulf of Mexico (Noirungsee et al., 2020). Interestingly, SCAs100 samples were clustered close to the in-situ samples; this is because the in-situ sediments of the Mariana Trench contained much higher amounts of short-chain alkanes (such as C_16_ and C_18_) but not long-chain alkanes [14]. Therefore, when SCAs or LCAs were added to the sediment samples, the similar microbial communities in the in-situ sediments grew rapidly in response to the SCA input, whereas different communities developed after the addition of LCAs.

After being incubated for 30 days, Gammaproteobacteria increased their relative abundance with time, and was the most abundant class in microbial communities with addition of SCAs and LCAs at 0.1 MPa (Figure 2), which is consistent with hydrocarbon-contaminated marine environments [12,36,70,71]. Many accumulating studies also demonstrated that Gammaproteobacteria usually increased their abundances in the oil-contaminated microcosms at 0.1 MPa, which had already been described as petroleum degraders [12,35,72,73,74,75,76], This result helps explain the enrichment of these bacteria in our study. However, the relative abundance of Gammaproteobacteria showed a decreasing trend when pressure was elevated to 100 MPa from 0 d to 150 days. These two opposite observations reflected that members of Gammaproteobacteria may be piezosensitive species with increasing pressure. Similar findings were also observed in a recent study investigating the effect of high pressure (22 MPa) on surface-water microbial communities [30].

In contrast, Alphaproteobacteria and Bacteroidetes showed an increase in relative abundance with incubation time (0–150 days) in 100 MPa-treated groups, whereas a decreasing trend was observed in these two phyla with time (0–30 days) at 0.1 MPa incubation (Figure 2). Likewise, Bacteroidetes reached the highest relative abundance in oil-contaminated surface waters when simulating deep-sea conditions, i.e., 22 MPa and 4 °C [30]. Several Bacteroidetes species also dominated hydrocarbon-degrading communities in sediment samples at 30 MPa (Luis et al., 2018).

At the genus and ASV levels, eight ASVs related to *Thalassolituus* almost dominated the gammaproteobacterial community involved in anaerobic hydrocarbon treatments at 0.1 MPa, with 77.1–87.9% of the average relative abundance (compared to 7.3% of the in-situ samples) (Figure 2, Figure 3, Figure 4 and Figure 5). However, *Thalassolituus* appeared to disappear (less than 1% of the relative abundance at 150 d) when pressure increased to 100 MPa (Figure 2, Figure 3, Figure 4 and Figure 5), suggesting that this species might be piezo-sensitive and could not resist extremely high pressure, as the abrupt changes in pressure could change bacterial physiology and often lead to cellular lysis [77]. Meanwhile, LDA analysis also showed that *Thalassolituus* was the most important microbial indicator responsible for community differences at 0.1 MPa (Figure 4). *Thalassolituus* is a typical marine strain of obligate hydrocarbonoclastic bacteria (OHCB) and grows almost exclusively on aliphatic hydrocarbons [72]. *Thalassolituus* was the most dominant alkane degrader over a range of chain-length *n*-alkanes (C_10_–C_32_) degradation in the estuary [31] and marine oil pollution environments [78,79]. Previous reports have also shown that *Thalassolituus*-related species are dominant in crude oil biodegradation, confirming the important role this taxon plays in oil enrichment [11,12,80,81,82,83]. 

Two important genera, *Idiomarina* and *Arenibacter*, increased their abundances to their maximum values (16.9% and 9.6%, respectively) in the anaerobic SCA enrichments at 100 MPa after being incubated for 150 days and seemed to play a major role in these treatments (Figure 2, Figure 3, Figure 4 and Figure 5). Recent relevant studies reported that *Idiomarina* can degrade polycyclic aromatic hydrocarbons (PAHs), crude oil, diesel oils, hydrocarbons, and biphenyl in various environments [30,84,85,86,87,88,89,90]. On the other hand, previous studies showed that *Arenibacter* strains were demonstrated to have the potential to degrade crude oil and PAHs, acting by producing biosurfactants or bioemulsifiers to increase the bioavailability of hydrocarbons [91,92,93,94]. However, these studies were only conducted at atmospheric pressure and temperatures of 21–28 °C; the enrichments of *Arenibacter* with the addition of *n*-alkanes in the sediments of the Mariana Trench at in-situ pressure and temperature were not reported. Together, our study implied that *Idiomarina* and *Arenibacter* may have the potential to utilize a variety of low-molecular-weight alkanes at 100 MPa and supply carbon and energy to meet their growth requirements. 

### 4.2. Effect of Different Hydrocarbon Components on Microbial Diversity and Community Structure

The molecular mass and structure of the hydrocarbons affect the rate and extent of their biodegradation; as the molecular mass, ring number, and alkyl-branching increase, the degradation processes become slower [25,95]. The in-lab simulations of the deep-sea plume demonstrated that 6–13 carbon alkanes were degraded first, with half-lives of 6–7 days [96]. We found that two different components of *n*-alkanes would influence the composition of the microbial community, and specific microbes may dominate or be outcompeted in the hydrocarbon enrichments [31,32,97].

Our study showed that the different molecular masses of *n*-alkanes may affect the microbial participants of their enrichments. In our study, the Shannon index of SCA treatments was always higher than that of LCA treatments, irrespective of pressure and oxygen (Figure S1 and Table S2), which suggested that microbes may favor the simple short-chain alkanes but not the long-chain alkanes in any case. On the other hand, compared to SCA treatments, three key gammaproteobacterial genera, including *Halomonas*, *Idiomarina*, and *Methylophaga*, were the predominant taxa for anaerobic LCA enrichments at 100 MPa and obviously increased their relative abundances (61.2%, 10%, and 6.0%, respectively) with the addition of LCAs incubated for about 150 days (Figure 2, Figure 3, Figure 4 and Figure 5). These results may suggest that *Halomonas*, *Idiomarina*, and *Methylophaga* had the special abilities to resist extremely high hydrostatic pressure and low temperature and may have had the related enzymes to degrade and utilize various high-molecular-weight alkanes (C_18_–C_36_) at 100 MPa/4 °C under anaerobic conditions. 

Many related investigations found that *Halomonas* was frequently involved in hydrocarbon degradation and produced an amphiphilic exopolymeric substance (EPS) for emulsifying hydrocarbons [75,98,99,100]. *Halomonas* have been reported to degrade PAHs in marine sediments [57,101,102], hypersaline habitats [87,103], California lake [104], and the Persian Gulf [86]. *Halomonas* could also degrade complex hydrocarbons such as diesel and crude oil in psychrotrophic habitats, including Antarctica [105] and the oil-contaminated deep ocean [12]. Nevertheless, few reports revealed that the *Halomonas* genus could utilize hydrocarbon under high hydrostatic pressure conditions [106].

Meanwhile, *Methylophaga* was strictly aerobic and moderately halophilic and showed an exclusive requirement for C_1_ sources (methanol, methylamine, and dimethylsulfide) as the sole carbon and energy source [107]. *Methylophaga* was observed to be involved in the enrichment of high molecular-weight PAHs [108,109], high-molecular-weight hydrocarbons [74,110,111], and crude oil [94]. 

### 4.3. Effect of Oxygen Concentration on Microbial Community Structure of n-Alkanes Enrichment Cultures

Oxygen concentrations exert important influences on the biodegradation of *n*-alkanes. This is due to the fact that aerobic alkane degraders use oxygen as a reactant for alkane molecule activation, whereas anaerobic alkane degraders use nitrate or sulfate as an electron acceptor [5]. The processes of aerobic and anaerobic alkanes degradation were obviously distinct, therefore, the main microbial communities responsible for aerobic and anaerobic hydrocarbon degradation were completely different. 

In this study, we found that oxygen concentration significantly affected the microbial community with *n*-alkane input at 100 MPa/4 °C based on nMDS analysis (Figure 1) and hierarchical cluster analysis (Figure S2). Most importantly, compared to the corresponding anaerobic treatments, Actinobacteria (e.g., *Microbacterium*) and Alphaproteobacteria (such as *Sulfitobacter* and *Phenylobacterium*) were the most abundant phyla (genera) responsible for aerobic hydrocarbon enrichments at 100 MPa (Figure 2, Figure 3, Figure 4 and Figure 5). 

At present, most incubation experiments on Actinobacteria are conducted at atmospheric pressure and room temperature. For instance, some actinobacterial isolates and their production peroxidases were demonstrated to play a key role in the processes of crude oil [112] and petroleum hydrocarbon biodegradation [17]. On the other hand, other actinobacterial genera were able to degrade efficiently high molecular weight PAHs [113,114,115], and medium and long chain *n*-alkanes (C_12_–C_20_–C_24_–C_30_) [116]. In our study, the relative abundance of Actinobacteria increased from almost 0.0% in in-situ sediments to 7.0–13.1% in aerobic *n*-alkane treatments at 100 MPa (Figure 2), which suggests that Actinobacteria may adapt well to 100 MPa and may play a critical role in aerobic *n*-alkane utilization. To our knowledge, this is the first report about members of Actinobacteria possibly involved in hydrocarbon degradation and utilization at extremely high pressure (100 MPa).

Only one genus of *Microbacterium*, affiliated with the Actinobacteria phylum, reached its maximum abundance of 6.9–13.0% in the aerobic *n*-alkane incubation at 100 MPa after 150 days (Figure 2, Figure 3, Figure 4 and Figure 5), which implied that *Microbacterium* had the potential to utilize hydrocarbons, especially short-chain alkanes. A series of related studies also revealed that *Microbacterium* could degrade hydrocarbons [117,118], crude oil [119], and PAHs [120,121,122,123,124] in various environments, such as hydrocarbon-contaminated soil and estuarine sediments. Nevertheless, these studies were performed at room temperature and did not involve hydrostatic pressure.

The relative abundance of *Sulfitobacter* increased to 8.2–14.5%, becoming the second abundant taxon in the aerobic *n*-alkanes incubation at 100 MPa (Figure 2, Figure 3, Figure 4 and Figure 5). This result was consistent with previous studies. For example, *Sulfitobacter* has been identified as a degrader of crude oil [25,73,125,126,127,128,129], hydrocarbons [130,131], and petroleum [30] in all kinds of environments. In addition, the draft genome sequences of two *Sulfitobacter* sp. revealed the presence of genes on their genomes involved in aromatic hydrocarbon enrichments [132]. However, only a few reports revealed that *Sulfitobacter* sp. was the most abundant species when enriching the surface-water samples with crude oil at 22 MPa. 

The relative abundance of *Phenylobacterium* increased to 11.1–17.9%, which became the top 3 dominant genera in the aerobic hydrocarbon enrichments at 100 MPa after 150 days (Figure 2, Figure 3, Figure 4 and Figure 5). A previous study showed that a new strain of *Phenylobacterium* was isolated and identified as petroleum-hydrocarbon-degrading bacteria from the contaminated soil [133,134,135]. *Phenylobacterium* is generally capable of degrading PAHs from crude oil-contaminated soils [136,137,138,139]. DNA-SIP technology revealed that *Phenylobacterium* was active benzo [a] pyrene (BaP) degrader in the BaP-contaminated soils [140]. Our results may reveal that *Phenylobacterium* increased may have the special abilities to utilize hydrocarbon and supply their growth and respiration requirements. 

## 5. Conclusions

Our study ingeniously simulated how the indigenous sediment microbial communities of the Mariana Trench responded to the SCAs or LCAs input under anaerobic or aerobic conditions at 0.1 MPa/100 MPa and 4 °C after long-period culture. Primarily, hydrostatic pressure and alkane components obviously affected microbial diversity; a higher microbial diversity was observed at 100 MPa than at 0.1 MPa. Meanwhile, microbial diversity in SCA treatments was always higher than that of LCA treatments. Furthermore, hydrostatic pressure and oxygen significantly affect the microbial community in the SCAs and LCAs enrichment cultures. For example, Gammaproteobacteria was the most abundant class for both anaerobic SCAs and LCA enrichments at 0.1 MPa, whereas Alphaproteobacteria and Bacteroidetes increased their relative abundance with anaerobic hydrocarbon input at 100 MPa. At 100 MPa, the most abundant phyla responsible for aerobic SCA and LCA enrichments were Actinobacteria and Alphaproteobacteria. These findings suggested that the sediment microorganisms may be important *n*-alkane degraders in the Mariana Trench. Our study further revealed the profound influence of pressure, oxygen, and hydrocarbon components on unique alkane-enriched microorganisms in the sediments of the Mariana Trench, especially under in-situ pressure and temperature. Future research may focus on the physiological and metabolic potential of these alkane-related microorganisms.

## Data Availability

All raw sequence datasets of 16S rRNA genes from this study have been deposited into the NCBI Sequence Read Achieve (SRA) database with accession no. PRJNA930158.

## Supplementary Materials

The following supporting information can be downloaded at: https://www.mdpi.com/article/10.3390/microorganisms11030630/s1, Figure S1: The temporal variations of Shannon index in microbial communities with aerobic and anaerobic n-alkanes enrichments at 0.1 and 100 MPa in the sediments of Mariana Trench; Figure S2: Hierarchical cluster analysis of microbial communities for aerobic and anaerobic hydrocarbon enrichments at 0.1 and 100 MPa in the sediments of Mariana Trench; Table S1: The ASV table of 55 samples in this study; Table S2: The sample ID, the number of reads and ASVs, Shannon and Simpson diversity indices, and value of Coverage in the 55 samples.
