# Supplementary material for: Influence of Extremely High Pressure and Oxygen on Hydrocarbon-Enriched Microbial Communities in Sediments from the Challenger Deep, Mariana Trench
Source: Microorganisms. 2023 Mar 1;11(3):630. doi: 10.3390/microorganisms11030630 (PMC10052102; doi:10.3390/microorganisms11030630)
Supplement: Supplementary file 1 [file microorganisms-11-00630-s001.zip › Figures S1 and S2.pdf]

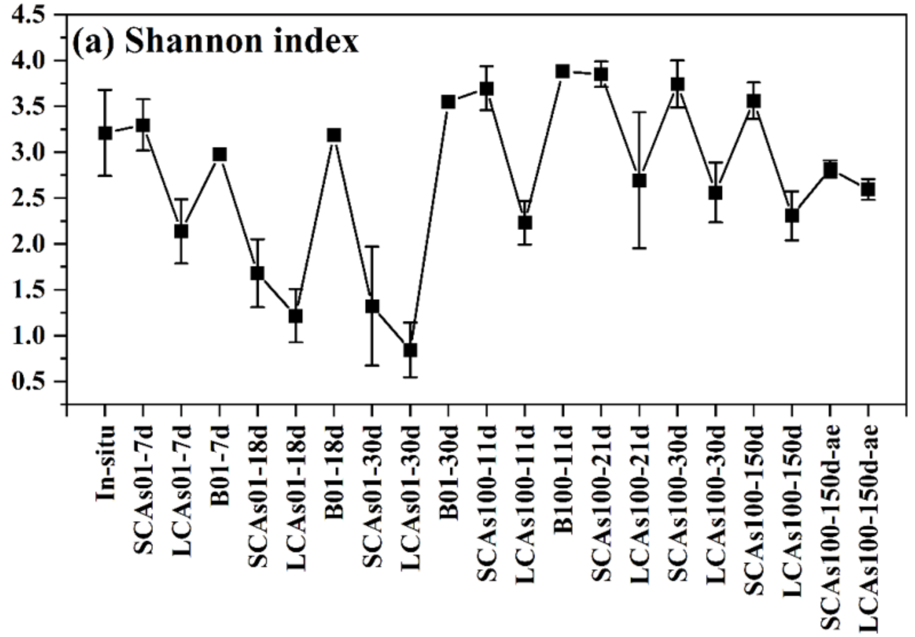

Figure S1. The temporal variations of Shannon index in microbial communities with aerobic and anaerobic n-alkanes enrichments at 0.1 and 100 MPa in the sediments of Mariana Trench.

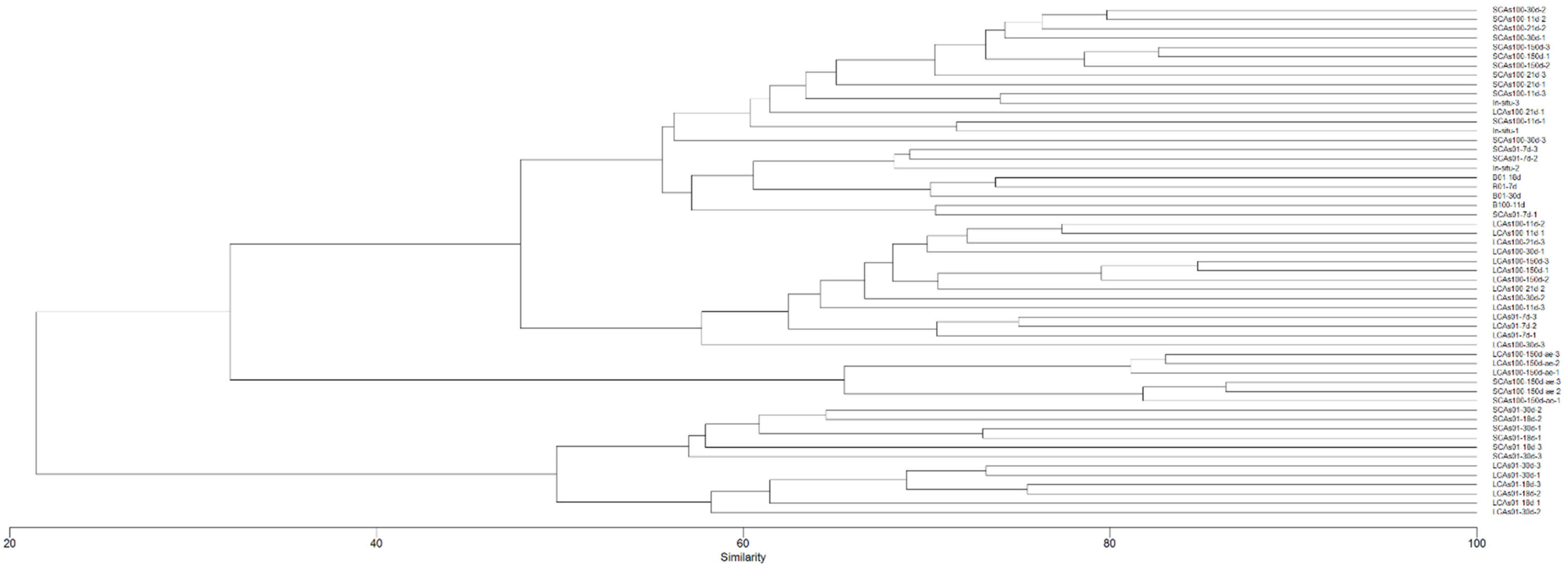

Figure S2. Hierarchical cluster analysis of microbial communities for aerobic and anaerobic hydrocarbon enrichments at 0.1 and 100 MPa in the sediments of Mariana Trench.
